# Bayesian Dynamical Modeling of Fixational Eye Movements

**DOI:** 10.1007/s00422-025-01010-8

**Published:** 2025-06-09

**Authors:** Lisa Schwetlick, Sebastian Reich, Ralf Engbert

**Affiliations:** 1 Department of Psychology, University of Potsdam, Potsdam, Germany; 2 Institute of Mathematics, University of Potsdam, Potsdam, Germany; 3 Research Focus Cognitive Sciences, University of Potsdam, Potsdam, Germany; 4 École Polytechnique Fédérale de Lausanne, Lausanne, Switzerland

**Keywords:** Fixational eye movements, Visual fixation, Physiological drift, Microsaccades, Self-avoiding walk, Bayesian data assimilation

## Abstract

Humans constantly move their eyes, even during visual fixations, where miniature (or fixational) eye movements occur involuntarily. Fixational eye movements comprise slow components (physiological drift and tremor) and fast components (microsaccades). The complex dynamics of physiological drift can be modeled qualitatively as a statistically self-avoiding random walk (SAW model, Engbert et al., 2011). In this study, we implement a data assimilation approach for the SAW model to explain statistics of fixational eye movements and microsaccades in experimental data obtained from high-resolution eye-tracking. We discuss and analyze the likelihood function for the SAW model, which allows us to apply Bayesian parameter estimation at the level of individual human observers. Based on model fitting, we find a relationship between the activation predicted by the SAW model and the occurrence of microsaccades. The model’s latent activation relative to microsaccade onsets and offsets using experimental data lends support to the existence of a triggering mechanism for microsaccades. Our findings suggest that the SAW model can capture individual differences and serve as a tool for exploring the relationship between physiological drift and microsaccades as the two most essential components of fixational eye movements. Our results contribute to understanding individual variability in microsaccade behaviors and the role of fixational eye movements in visual information processing.

The human eye is never truly at rest. At a macro-level the eyes move in a sequence of fixations and saccades (Schwetlick et al. 2020), moving different aspects of the visual world into the receptor-dense center of the visual field (i.e., the fovea). Despite the misleading term, the eyes are far from stationary during fixations (Ditchburn et al. 1959; Kowler 2011; Martinez-Conde et al. 2004; Rucci and Victor 2015; Poletti 2023). Microscopic or fixational movements constantly shift the visual input over the receptors in the retina. Three components of fixational eye movement behavior are distinguished: a slow random motion called physiological drift, a high-frequency tremor, and high-velocity microsaccades (Alexander and Martinez-Conde 2019; Ciuffreda and Tannen 1995). The comparatively small amplitude of tremor can not be resolved using current video-based eye tracking, is likely too small to have meaningful visual consequences (Bowers et al. 2019), and is therefore neglected in the following.

The function of fixational eye movements, their underlying mechanisms, as well as the consequences for processing in the visual system have yet to be fully understood. While fixational eye movements are found ubiquitously across species and in all primates (Ko et al. 2016), The generation of fixational eye movements varies between individuals and is highly characteristic (Engbert and Kliegl 2003, 2004; Cherici et al. 2012; Poynter et al. 2013). To model the individual spatial statistics of physiological drift and its relationship to microsaccades, we briefly discuss the two movement components.

During physiological drift, the eyes move in a pattern that resembles Brownian motion (Engbert and Kliegl 2004; Engbert 2006; Burak et al. 2010; Pitkow et al. 2007), i.e., eye position meanders stochastically with increasing variance over time. However, a more detailed analysis indicates that fixational eye movements represent an interesting example of fractional Brownian motion (Mandelbrot and Van Ness 1968; Metzler and Klafter 2000). A corresponding analysis (Collins and De Luca 1995) can be carried out by computing the mean square displacement (MSD) at different time lags (Engbert and Kliegl 2004; Herrmann et al. 2017). In Brownian motion, the MSD increases linearly with time lag. In fixational eye movements, however, a superdiffusive tendency is found over short time scales ($\lesssim 50$ ms), which is also referred to as persistence. Over longer time scales ($\gtrsim 100$ ms) physiological drift is found to be anti-persistent. We interpret such behavior by assuming that fixational eye movements maximize movement over short time scales to counteract retinal fatigue while reducing variance over a long time scale to maintain visual fixation at an intended region of interest or object. (Engbert and Kliegl 2004).

Microsaccades share their kinematic properties with their larger counterparts, such as acceleration profile, main sequence (i.e., the linear relationship between log amplitude and log peak velocity; see Bahill et al. (1975)), and are generated by the same neural circuits in the brainstem (Hafed et al. 2009, 2021). Microsaccades are distinguished mainly by their smaller amplitude, usually thresholded at $<1^\circ$ (Martinez-Conde et al. 2004; Poletti 2023). Microsaccades occur at a rate which is highly variable between subjects (Engbert and Kliegl 2003; Martinez-Conde et al. 2004). Attentional mechanisms also modulate microsaccades in both their rate and orientation Hafed and Clark (2002); Engbert and Kliegl (2003); Yuval-Greenberg et al. (2014). The rate variation is induced by display changes and modulated by ongoing cognition: First, a reduced microsaccade rate follows target onset, and then an increased rate (Engbert and Kliegl 2003) is observed. Microsaccade orientations are modulated by covert attention, e.g., during spatial cueing (Hafed and Clark 2002; Engbert and Kliegl 2003). The general patterns of interactions of microsaccade rates and orientations is more complex (Engbert 2006), however, a computational models has been proposed (Engbert 2012).

Early accounts of fixational eye movements conceptualized them as the result of random firing from oculomotor units (Eizenman et al. 1985), or else as a nuisance component that causes blurring, if not corrected by the visual system (Packer and Williams 1992; Burak et al. 2010). More recent evidence points to fixational eye movement being a necessary and useful component of visual exploration in counteracting receptor adaptation (Rucci and Victor 2015; Engbert and Mergenthaler 2006; Intoy and Rucci 2020; Poletti 2023). First, fixational eye movements prevent visual fading (Ditchburn et al. 1959; Martinez-Conde et al. 2006) caused by neural adaptation (Coppola and Purves 1996; Martinez-Conde et al. 2004, 2006). Although fading prevention may be achieved by drift alone, microsaccades are much more effective at restoring vision after fading has set in (McCamy et al. 2014, 2012). Second, both drift (Rucci and Victor 2015; Boi et al. 2017) and microsaccades (Poletti et al. 2013) have been found to facilitate high acuity pattern vision (Intoy and Rucci 2020). Specifically, the performance of an edge detection model can be improved by the addition of a movement component (Schmittwilken and Maertens 2022). In another study, Anderson et al. (2020) use a Bayesian model of neurons during early visual processing that simultaneously estimates eye motion and object shape. This study also reports that drift motions benefit high-acuity vision, mainly by averaging over the inhomogeneities in the retinal receptors and receptor density. Finally, microsaccades and drift have been found to be both corrective and exploratory. Most microsaccades, especially in a high precision environment, are corrective, returning the eyes to the intended fixation point (Tian et al. 2016, 2018; Buonocore et al. 2021). However, when peripheral regions contain coincident neuronal activity at the moment of microsaccade triggering, the movement can instead shift away from fixation. In these cases, microsaccades serve a more exploratory or error-producing role by bringing new visual details into the center of the field (Engbert and Kliegl 2004). Microsaccades are typically preceded by a reduction in drift (Engbert and Mergenthaler 2006; Sinn and Engbert 2016), supporting the idea that the phenomena are functionally related. However, Chen and Hafed (2013) observed a transient increase in retinal image slip following microsaccades, indicating that their interaction may be more complex. The current study’s research goal is to analyze the relationship between slow fixational eye movement components and microsaccades based on quantitative modeling while taking into account individual differences observed in experimental data.

**Fig. 1:**
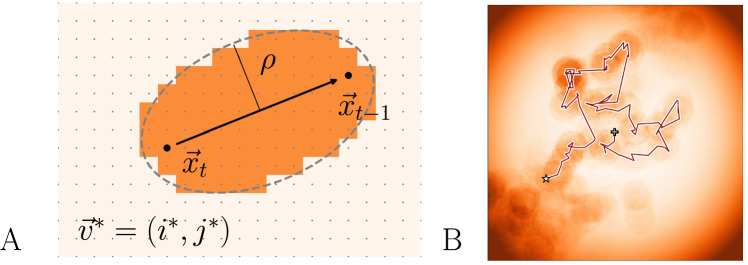
**A** Schematic of how activation is generated within the self-avoiding walk (SAW) model. Lattice points within a distance $\rho$ from the eye’s trajectory are assigned increased activation, forming a trace of the path taken. This trace is used by the model to avoid recently visited areas. **B** Simulated fixational eye movement trajectories, illustrating the SAW model. Starting from the position marked by the + and ending at the position marked by the star, the model generates activation along the trajectory. The movement is constrained by an activation potential centered around the fixation position. Note that in order to avoid starting from a blank activation map we initialize a background activation profile by using the model to simulate some random steps. This causes some activation to be visible at locations that have not been visited. Activation decays gradually over time

The SAW model integrates several of the above properties of fixational eye movement and is biologically plausible (Engbert et al. 2011; Engbert 2012; Herrmann et al. 2017). The model describes physiological drift by assuming a **S**elf-**A**voiding (random) **W**alk (SAW) confined in a movement potential that limits the movements to reproduce visual fixation. A random walk on a lattice represents the trajectory of the eye. As the random walk traverses the lattice, visited locations are activated (see Figure 1). The activation represents the memory process that keeps track of the recently visited locations (Freund and Grassberger 1992). This generative model successfully reproduces both the persistent and anti-persistent statistical properties of ocular drift Engbert et al. (2011). An extension of the model (Herrmann et al. 2017) implements neurophysiological delays, thereby matching the characteristic oscillations found in the displacement autocorrelation. Within the SAW model framework, Engbert et al. (2011) proposed a mechanism for generating microsaccades based on the activation in the SAW model.

We developed the SAW model (2011) as a qualitative model that produces key properties of fixational eye movements and microsaccades. Therefore, we worked with a relatively coarse resolution (a 51$\times$51 grid) and drift steps of size $\pm 1$ units. The current paper extends the model to a higher spatial resolution (100$\times$100) with quasi-continuous drift steps. Furthermore, we implement numerical computation of the model’s likelihood function. These modifications permit the fitting of experimental data in a fully Bayesian framework of parameter inference. We estimate model parameters for individual observers and find that the estimated parameters represent individual spatial statistics of physiological drift. Based on the quantitative agreement between simulated and experimental drift movements, we explore the relation to microsaccades. We use the model’s latent activation to investigate potential mechanisms for triggering microsaccades. Since fixational eye movements strongly impact the spatiotemporal input that the visual system processes, reproducing these movements from a computer-implemented mathematical model is essential for a better understanding of visual functioning.

## Results

In the first step, we define the SAW model and describe the computation of its likelihood function. In the second step, we use the likelihood computation to estimate parameters for individual human participants. Finally, we use the model to generate data and conduct posterior predictive checks and exploratory analyses concerning the relationship between drift and microsaccades.

### The model

As a theoretical starting point, the SAW model generates a random walk which is statistically self-avoiding (Freund and Grassberger 1992). The self-avoiding walk is implemented on an $L \times L$ lattice where nodes are given by (*i*, *j*) with $i,j=1,2,3,\ldots ,L$. Each node carries some activation $a_t(i,j)$ at time *t*, which can be interpreted as neural firing rates. Initial activation values are set to $a_{t=0}(i,j) = 10^{-1}$ for all (*i*, *j*). At time $t=1,2,3,\dots$, first, the current activation of each node (*i*, *j*) across the field decays, according to

1 $$\begin{aligned} a_{t+1}(i,j) = \epsilon \cdot a_{t}(i,j) \,, \end{aligned}$$

where $\epsilon = 1 - (10^{\gamma })$, representing the speed of the process memory decay. Second, activation is added to the nodes along the walker’s trajectory, i.e.,

2 $$\begin{aligned} a_{t+1}(i^\star ,j^\star ) = a_t(i^\star ,j^\star ) + 1 \;. \end{aligned}$$

Next, we implement a rule for activating lattice positions $(i^\star ,j^\star )$ along the trajectory. We define an ellipse which is drawn such that the positions at times *t* and $t+1$ are the foci of the ellipse. The parameter $\rho$ represents the size of the minor axis of the ellipse; the numerical value is set to 12 units (see Fig. 1). The lattice positions $\vec {v}^\star = (i^\star ,j^\star )$ are defined as all lattice sites within the ellipse, which are activated according to Eq. (2).

The discretized map $\{a_t(i,j)\}$ of neural activations can be interpreted biologically, since grid cells have been found in entorhinal cortex which keep track of previously visited locations (Killian et al. 2012).

In principle, the self-avoiding walk can produce persistent motion if parameters are selected appropriately. However, during visual fixation, human observers are able to keep the eyes at an intended target. Therefore, the model implements a movement potential, which confines the random walk and represents a mechanism of fixation control. As a consequence, the model can produce anti-persistent motion on the longer time scale. Thus, the self-avoiding motion in a potential could maintain fixation at the intended location despite the necessity of refreshing the retinal input. The (time-independent) confining potential *u* is centered in the lattice and takes the form

3 $$\begin{aligned} u(i,j) = \lambda \frac{\left( \sqrt{(i-\frac{L}{2})^2+(j-\frac{L}{2})^2}\right) ^\nu }{L^\nu } \;. \end{aligned}$$

Within this potential, motion is controlled by the sum of the self-generated activation $a_t(i,j)$ and the potential *u*(*i*, *j*), i.e.,

4 $$\begin{aligned} q_t(i,j)=a_t(i,j)+u(i,j) \end{aligned}$$

In order to choose the next step of the walker, we compute the probability of the next eye position as $\pi _n$, consisting of $q_t(ij)$, self generated activation and potential, and a time-independent stepping distribution, which controls the size of the movements, i.e.,

5 $$\begin{aligned} \pi _t(i,j) = {q_t(i,j)^{-\eta }}\, \exp \left( -\left[ \left( \frac{i}{r_i}\right) ^\phi + \left( \frac{j}{r_j}\right) ^\phi \right] \right) \;. \end{aligned}$$

From $\pi _t(i,j)$ we select the eye position at time $t+1$ using a linear selection algorithm.

As a result, each time step comprises the following sequence: first, relaxation of the current activation; second, selection of the following eye position under consideration of a stepping distribution; and third, an increase of the activation values along the current trajectory. Several free parameters control our model’s behavior. In this study, we selected a subset of parameters for estimation, namely $\gamma$, the speed of the relaxation, the size of the stepping distribution $r_i$ and $r_j$, the slope of the stepping distribution $\phi$ and the slope of the potential $\lambda$ (i.e., $\theta = [\gamma , r_i, r_j, \phi , \lambda ]$). We set $\rho = 12$, $\nu = 3$, and $\eta = 1$ to constrain the model and obtain numerically stable behavior during parameter estimation. The parameters selected for estimation are of primary interest, as their values are interpretable quantities that may give insight into the biological plausibility of the model.

### Likelihood function: sequential computation

The observation that makes the model compatible with a likelihood-based approach is the fact that the likelihood *L* at each time for each location on the lattice is given by the selection map $\pi$. In other words given the time-ordered data $X_t=\{x_1,x_2\dots ,x_t\}$, we can calculate the probability of observing the walker in position *x* at time *t* given the model and given all previous positions $X_{t-1}$, i.e. $P_{\mathrm M}\left( x_t \mid X_{t-1}, \theta \right)$. The likelihood of a sequence of *t* events (Schütt et al. 2017) is therefore given by the product of *t* conditional probabilities $\mathcal{L}_M(\theta |X_t)$, which is given by

6 $$\begin{aligned} \mathcal {L}\left( \theta \mid X_n\right) = P_{\mathrm M}\left( x_1 \mid \theta \right) \prod _{i=2}^n P_{\mathrm M}\left( x_t \mid X_{t-1}, \theta \right) \;. \end{aligned}$$

In order to estimate the parameters of the model, we use Bayes’ theorem to compute the probability of the parameters $\theta = [\gamma , r_i, r_j, \phi , \lambda ]$ given the data as

7 $$\begin{aligned} P(\theta \mid X_n) = \frac{L_{\mathrm M}(\theta \mid {X_n})P(\theta )}{\int _\Theta L_{\mathrm M}(\theta \mid X_n)P(\theta ){\mathrm d}\theta } \;. \end{aligned}$$

A large literature exists to solve likelihood-based parameter estimation computationally. In order to leverage the full power of the likelihood-based approach we estimate the full Bayesian posteriors for each parameter using a differential evolution adaptive metropolis sampling algorithm (DREAM) (Laloy and Vrugt 2012; Shockley et al. 2018). More details are provided in the Methods section.

### Parameter estimation results

**Fig. 2:**
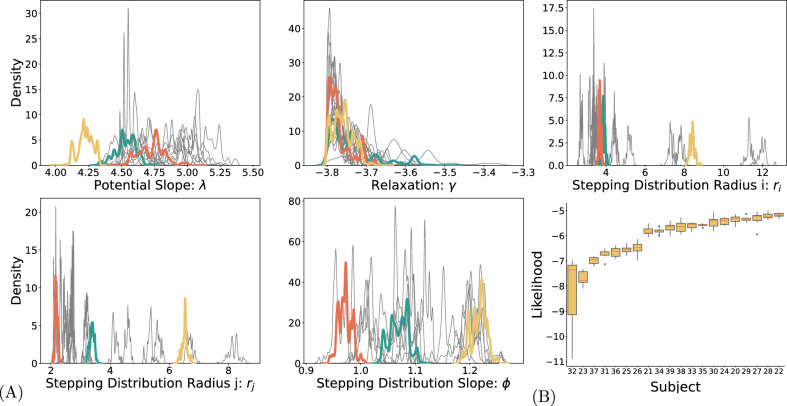
(**A**) Marginal posteriors for the five estimated parameters; the grey lines represent different participants, colored lines highlight three participants for comparison. For all five parameters, the posteriors converged to distinct peaks for the different participants. (**B**) Box plot showing the total log-likelihood of the model fit across all trials, separately for each participant. Participants are sorted from lowest to highest based on their average log-likelihood. This shows how well the model fits the data for each person

We estimated the values of the free parameters of the SAW model for each subject independently based on data. The chosen priors (see Appendix for details) were relatively uninformative truncated Gaussians. Figure 2 A presents the marginal posteriors for each participant in the study. To assess how well the model captured each participant’s behavior overall, we computed the total log-likelihood across all test trials. Figure 2B shows these values sorted by participant, revealing this variation in model fit across individuals, with some participants consistently showing better fits than others.

In the Appendix (Table 2) we present the point estimates and 98% confidence intervals (Kruschke 2014) for each parameter.

**Fig. 3:**
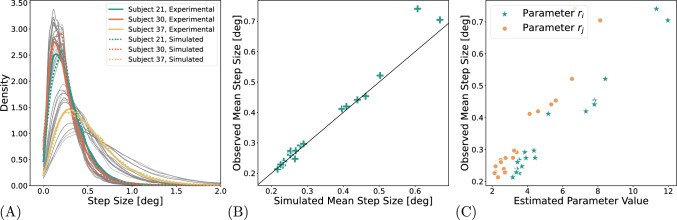
The fit between step sizes in the model and in the data. (**A**) Step size distributions for experimental (solid lines) and simulated (dashed lines) data. Each line represents one participant. Colored lines highlight three individuals for clarity; gray lines show the rest. This panel illustrates how well the model captures individual differences in step size. (**B**) shows the correlation between mean step size in the experimental data and in the simulated data. Each cross represents the experimental and fitted mean step size for one participant. (**C**) shows the correlation between observed step size and the fitted model parameters $r_i$ and $r_j$, which determine the width of the stepping distribution. As expected, larger values of $r_i$ and $r_j$ correspond to larger step sizes in the data

Biologically plausible models are designed to be grounded in real-world biological mechanisms and processes. As such, the values of their parameters reflect the known properties of these mechanisms. It is therefore informative to investigate the parameter values themselves, as they permit inferences about concrete aspects of the data generating process. First, the parameters $r_i$ and $r_j$ correspond to the widths of the stepping distribution. The final selection map in the model (Eq. 5), and consequently the resulting predictions for step sizes, depend on the stepping distribution containing $r_i$ and $r_j$, as well as the confining potential and activation. We define the step size as the distance travelled from one measured experimental sample to the next, i.e., 2 ms as we are using a sampling rate of 500 Hz. As shown in Figure 3C the parameters $r_i$ and $r_j$ are strongly correlated with the empirical step size distribution.

The parameter $\phi$ represents the slope of the stepping distribution. Higher values are associated with a stronger tendency to move along the cardinal directions. The estimated values for $\phi$ range from 0.9 to 1.3, indicating that the preference for cardinal directions is stronger in some participants than in others. The model captures individual differences in the stepping distributions, as demonstrated by the distinct posteriors obtained for $\phi$, $r_i$ and $r_j$.

Next, the parameter $\gamma$ relates to the speed of memory decay in the process, where smaller, more strongly negative values cause slower decay, i.e., longer memory of the visited locations, and larger values closer to 0 cause faster memory decay, i.e., shorter memory. It was bounded in the estimation to a minimum of $-4$, as smaller value, i.e. less relaxation, caused the activation in the system to continuously increase, making the model numerically unstable. We find an average value of $\gamma$ of 3.75 which indicates that activation decreases by 25% over the course of a trial duration of 3 s. Parameter $\gamma$ accounts for relatively small individual variability. For most of the participants, estimates indicate a long memory for activation that represented the past trajectory in the system.

Finally, parameter $\lambda$ represents the slope of the confining potential. The shape parameter of the potential function was fixed to 3, fixing the qualitative form to a steeper gradient than in a quadratic case (Engbert et al. 2011). The slope was considered a free parameter for the estimation. As the different participants’ data lead to different decay and stepping parameters, the slope of the potential needs to accommodate different resulting values of mean system activation.

### Posterior predictive checks

Using the estimated parameters (i.e., the posterior parameter distributions), we simulated artificial data sets of the same size as the experimental test data sets. We know that the model can, in principle, recreate statistical tendencies of ocular drift found in experimental data on a qualitative level (Engbert et al. 2011). Posterior predictive checks show that the fitted model reproduces the expected statistical tendencies, i.e., turning angle- and step size distributions, including inter-individual differences.

Fitted primarily by parameters $r_i$ and $r_j$, the step size distribution is represented in Figure 3 A. As defined above, the step size is the spatial distance between two subsequent samples. In order to condense the distribution of step sizes into one summary value, we computed the mean of each distribution. Thus, Figure 3 B shows the correspondence between the mean true and simulated step sizes. The model fits the step size very precisely and perfectly captures the differences in the individual preferred step size.

**Fig. 4:**
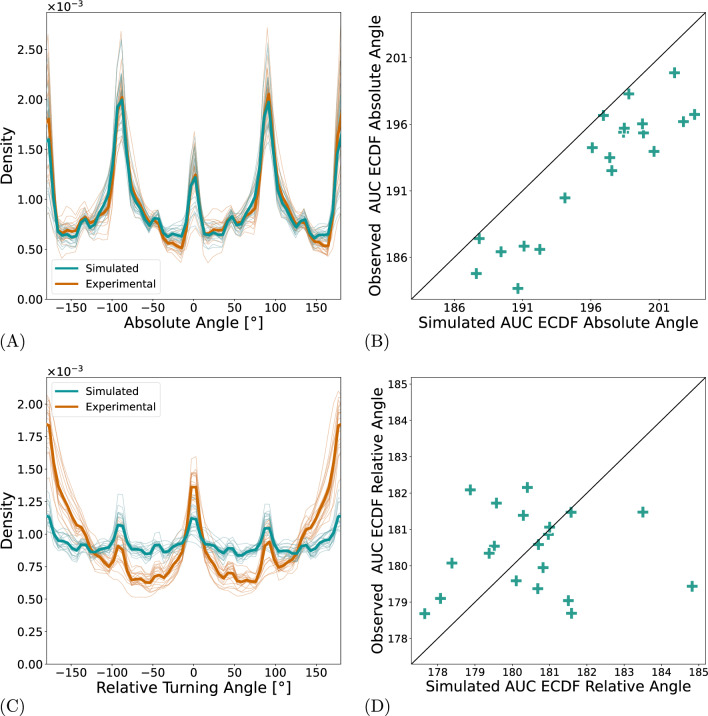
Turning angle distributions. (**A**) and (**C**) show the absolute and relative turning angles of one sample to the next. Thin lines represent individual subjects and thicker lines represent the means. Simulated data is shown in green while experimental data is red. Panels (**B**) and (**D**) show the respective correlations between simulated and experimental data. We characterize the angle distributions using the area under curve (AUC) of the empirical cumulative density function (ECDF). For details see the Methods section

Next, we investigated the absolute and relative turning angles. There exists a preference in both ocular drift and (micro)saccades for movements in the cardinal directions, which may be caused by the structure of the ocular muscles (Sparks 2002). This fact is captured well by the model (Figure 4A). The relevant parameter responsible for this effect is the stepping distribution slope $\phi$, which shapes the stepping distribution to have a stronger or weaker preference for the cardinal directions. In order to ascertain whether the differences in individual behavior can also be reproduced, we use the area under the cumulative density function as a summary statistic (see the Methods section). The result in Figure 4B shows that the model is capturing individual differences. Furthermore, there is also a tendency for movements to be in line with or orthogonal to the previous movement vector. While this tendency is a lot more pronounced in the experimental data than in the simulated data, the simulations do show a qualitative reproduction of the trend (Figure 4C). However, it is likely that the peaks are driven by the preference in absolute angles, as no mechanism for relative angles was built into the model. We propose that the difference between the simulated and experimental relative turning angle distributions reveals the part of the relative turning angle distribution that must be accounted for by an additional, independent model mechanism.

**Fig. 5:**
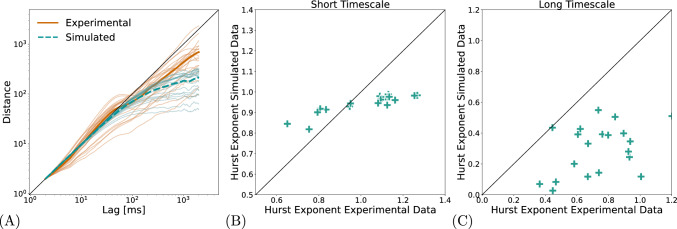
Persistent and Anti-persistent behavior. (**A**) shows the MSD of simulated and experimental data, where thinner lines represent individual subjects and thicker line represent the averages of experimental data in red and simulated data in green. Note that the lag is given in ms, whereas the distance is given in normalized units, in order to visualize the slopes in comparison to the identity line. (**B**) and (**C**) shows the correlation of simulated and experimental data of linear fits of the Hurst Exponents for the short and long timescale for individual subjects

Correlations in fixational eye movements can be studied by computed the mean-square displacement (MSD) as a function of the time lag between two data samples. Empirical ocular drift movements have been found to be persistent for small time lags, indicated by a slope $>1$ in double-logarithmic representation, and anti-persistent on longer timescales, i.e., slope $<1$ (Engbert and Kliegl 2004). In Figure 5A, the persistent component in our data is not pronounced for all participants, which is reported in earlier experimental studies (Herrmann et al. 2017). In Figure 5B, we plotted the slope parameter from simulations against experimental data for the short time scale (60 to 80 ms); in Figure 5C, the same analysis is done for the long time scale (100 to 1000 ms). While the model captures the general change from short to long time scales, the persistence is too weak at the short time scale and the anti-persistence is too strong on the long time scale.

Exploratory analyses of the model demonstrated that simulations can produce persistent and anti-persistent behaviors by varying the free parameters. Possible reasons why this trend was insufficiently captured by the fitted models are that the MSD is a less dominant tendency compared to other statistics and that our selection of free model parameters were correlated in a way that limited the ability to fit this particular tendency. Alternatively, the self-avoidance of the model may not be the only cause for early persistence, suggesting a model with an explicit exploration mechanism. Nonetheless the qualitative change in behavior is clearly present in the simulated data. Accordingly, the correlation between the experimental data and the simulated data show that the present model fits only account for a small part (roughly 10%) of the individual variation (5B and C).

### Investigating microsaccades

Previous work concerning the SAW model has suggested a connection between the self-avoiding properties of the random walk and microsaccade triggers. A reduction in movement tends to precede microsaccades (Engbert and Mergenthaler 2006). Following this reasoning, in the model, a decrease in movement corresponds to a build-up of activation in the current position. Thus, we investigated whether the activation predicted by the model is indeed related to the occurrence of microsaccades. Specifically, we calculated the activation $q_t(i,j)$ at times relative to microsaccade onsets $t_{\text {M}S-on}$ and offsets $t_{\text {M}S-off}$ using the experimental data.

**Fig. 6:**
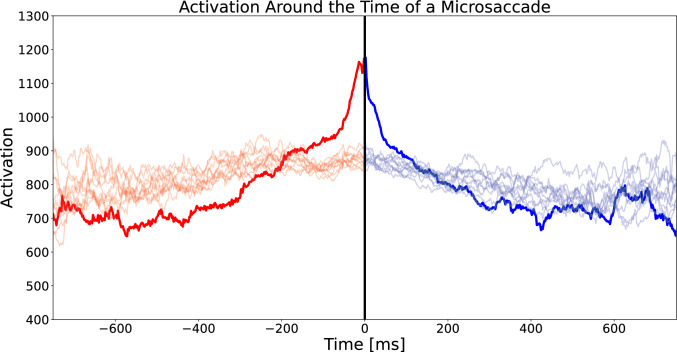
The average activation in the model around the time of a microsaccade. The less opaque lines represent randomized controls, where microsaccade onsets were randomized over the trials within one subject. In the data we find a distinct rise in activation before the time of a microsaccade and a drop in activation after

Figure 6 shows the average activation around the time of a microsaccade, which is consistent with the hypothesis that high activity levels are related to triggering microsaccades. To better understand the extent of the effect, we randomized the microsaccade onsets within each subject and computed the same trajectory on the randomized data. The activation rises more before a microsaccade and drops more steeply than the randomized controls.

**Fig. 7:**
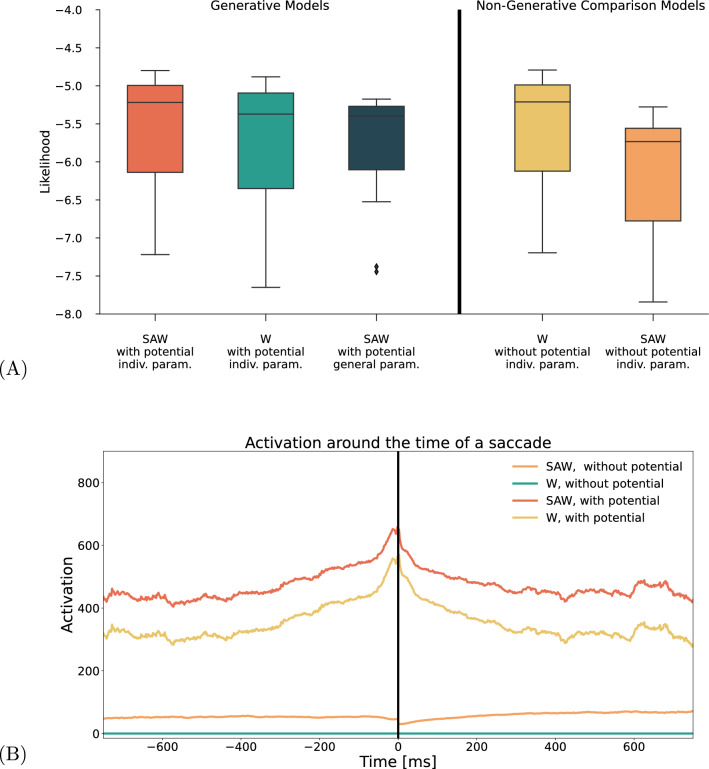
Comparisons of the different model versions. Panel A shows the 5 different versions of the model, including different components. We show that among biologically plausible models that the proposed mechanisms and the individual fitting procedure confer a likelihood benefit. Among Non generative models a pure random walk with fitted step sizes, while not biologically plausible, also achieves a high likelihood score. Panel C shows the model activation around the time of a saccade. The activation peak emerges only in the models that contain a confining potential

### Model comparisons

The proposed model comprises three main components: the random walk with a stepping distribution, the self-activated trace memory, and the potential. In theory, the combination of both creates an interplay between persistence and fixation control. In order to better understand the role of each component, we created 3 control models by removing individual components. Specifically we investigate

1. the full baseline SAW model, that contains all three components
2. a model that is a random walk in a potential (W),
3. a model that is a random walk without a potential(W-NP),
4. a model that is a random walk with self-avoidance but without a potential (SAW-NP).

Note that the latter two models differ from the first two significantly in that they are not generative and, therefore, are not biologically plausible. In the absence of a potential it is still possible to compute the likelihood, but it is not possible to usefully simulate data from them, as there is nothing stopping the walker from simply walking away. We include them here, because they provide a relevant comparison. However, these models must be treated as substantially different concerning the conclusions they permit.

First, we compare the models in terms of their likelihood (Figure 7A). Each model was evaluated on the test data set, using the same parameters wherever applicable. Our findings indicate that SAW outperforms the model version without self-activation (W). Removing the potential and maintaining self-avoidance (SAW-NP) also reduces performance. However, we also find that the non-generative model without either potential or self-avoidance (W-NP) produces an almost identical likelihood to SAW. This result suggests that individually fitted stepping distributions and general random walk behavior capture the data’s most predominant features. It is important to note that the likelihood is a very general measure of model performance – the model (W-NP) is neither biologically plausible nor can it capture any additional statistical properties of the data. Thus, the lesson learned from this modeling study is twofold: First, a high likelihood is not always a guarantee of an appropriate model (see also Schütt et al., 2017), and second, that either a potential or self-avoidance is not beneficial to model performance. Each component, added individually, actually reduces the model likelihood. The fact that their joint effect reestablishes a similar likelihood while simultaneously making the model more biologically plausible should be considered a success. We added another model to this comparison: the SAW model without individual parameter fits by subject. We observe that on average, fitting individual subjects is a distinct benefit. However, the averaged parameter model reduces high likelihood values and the number of very low likelihood values.

Second, we used our comparison models to investigate which components drive the microsaccade effect. The apparent picture in Figure 7 shows that the activation peak is present only for the two models with a potential. This finding indicates that the effect is not driven, as we supposed, by a build-up of self-generated activation but rather by the potential. This result is consistent with the idea that the microsaccades that drive the effect are related to the control of the fixation position.

## Discussion

Fixational eye movements display significant randomness, and their origin and purpose have been debated in the literature. Mathematical modeling approaches have contributed insights into the neurophysiological origin of the movement (Eizenman et al. 1985; Ben-Shushan et al. 2022), the desirable or undesirable consequences of the motion on image processing (Schmittwilken and Maertens 2022; Anderson et al. 2020), and the spatiotemporal statistics of the drift trajectories (Burak et al. 2010; Engbert et al. 2011; Roberts et al. 2013). We performed Bayesian likelihood-based parameter inference of a self-avoiding random walk model for fixational drift at the level of individual observers. The estimation of the parameters converges to distinct marginal posteriors, and data simulated based on the fitted models reproduces individually characteristic behavior. In the second step, we propose a relationship between the microsaccade rate and peaks in the model’s latent activation state. An exploratory, data-driven analysis confirms this intuition.

### Individual variability

A complex combination of factors, such as oculomotor control, attention, and cognition, controls fixational eye movements. The specific observed patterns vary significantly by individual both for measures of ocular drift (Cherici et al. 2012) and microsaccades (Poynter et al. 2013). Our results indicate that the individual variability in drift can at least partly be captured by the parameters of the SAW model. The average preferred step size is a particularly pertinent example ($r_i, r_j$), but also directional preferences ($\phi$) and potential slope ($\lambda$) are different between subjects. These parametrizations are sufficient to simulate data that mirrors the characteristic features of individuals. To our knowledge, this is the first paper to model individual variation of fixational eye movement trajectories.

The variability in drift between individuals is related to the individual variability in acuity (Clarke et al. 2021). Individual differences in fixational eye movement may be related to a range of factors, including the precise acuity of the eye and the tendency to maintain precise fixation (Cherici et al. 2012). Moreover, attentional preferences found in macroscopic eye movement during facial feature viewing translate to microscopic eye movement preferences (Shelchkova et al. 2019).

We find a distinct benefit from using individually fitted data sets over a single averaged value for each parameter. This benefit becomes apparent in better convergence properties of the parameter estimation, as the solid and distinct influences otherwise cause complex, multi-modal posteriors. Moreover, the individual fits cause a higher overall likelihood and fit of individual characteristics. However, while model based in averaged parameter values has a lower likelihood, it also reduces the number of very low likelihood data points. Thus, individuals whose data can not be easily fitted would benefit from a normative influence of other subjects. This observation suggests using a hierarchical Bayesian modeling approach in future studies.

### The confining potential

The characteristic Mean Square Displacement (MSD) of fixational drift is persistent at small time lags and anti-persistent at longer lags (Engbert and Kliegl 2004; Herrmann et al. 2017). This is consistently the case, when averaging over large amounts of data. On an individual level, however, this tendency is not always equally pronounced. While the SAW model reproduces the transition to anti-persistent behavior well, it does not adequately represent the persistent component, with the current data. As, in principle, self-avoiding random walk models can produce persistent behavior (Engbert et al. 2011; Roberts et al. 2013), this may be caused by a number of factors including the selection of fitted free model parameters, the relatively low persistence in the present data set, or a dominance of other statistical tendencies. Alternatively, it is possible that the strength of the persistent trend, which the SAW model frames as the result of self-avoidance, is in fact amplified by an additional explicit exploration mechanism which remains to be identified.

Exploration, or the explicit persistence of the trajectory, is highly variable, even between trials. The confining potential in the SAW model is static, representing a fixed intended fixation position. These assumptions are a simplification, as the intention may change over time. Experimentally, we find a large amount of variation in the cohesion of the drift. In some trials, drift consistently occurs around a specific position. In others, it is evident that two intended fixation positions coincided over the course of the trial. In others, drift consistently maintains its direction away from the starting point. As the task and stimulus in the experiment were the same for all trials, there is little evidence to explain this variation, aside from random variation or influence of recent past stimuli. A potential future direction for the model could be implementing a dynamic confining potential centered around a moving average of several recent samples. The limitation underscores the need for more comprehensive and accurate models to capture better the complex and individual characteristics of ocular drift behavior.

In addition to low-level constraints, recent studies show that drift can also be modulated by task demands and top-down expectations (Intoy and Rucci 2020; Lin et al. 2023). For instance, drift amplitude decreases during fine spatial discrimination, and its direction can reflect prior knowledge about target location. The current SAW model does not explicitly include mechanisms for volitional or anticipatory control. However, these effects could potentially be captured by making the potential dynamic or by biasing the stepping distribution based on task-relevant inputs. Incorporating such top-down influences is a promising avenue for extending the model to more complex, naturalistic viewing scenarios.

### The relationship between drift and microsaccades

Early hypotheses suggested that microsaccades serve a corrective function for ocular drift (Ditchburn and Ginsborg 1952; Cornsweet 1956; Nachmias 1959). Experimentally, however, no reliable correlation data confirms this hypothesis, as microsaccades can be explorative as well as corrective. Specifically, at shorter time scales, microsaccades induce persistent correlations, while at longer time scales, they tend to reverse the movement to correct the fixation position (Engbert and Kliegl 2004). Additionally, studies have shown that during high-acuity observational tasks, participants naturally suppress microsaccades without training (Bridgeman and Palca 1980; Winterson and Collewijn 1976), leading to the conclusion that microsaccades may serve no useful purpose (Kowler and Steinman 1980). However, more recent research has demonstrated that microsaccades can enhance the visibility of peripheral stimuli (Martinez-Conde et al. 2006), facilitate high-acuity vision (Poletti et al. 2013; Intoy and Rucci 2020), and are responsive to task demands (Ko et al. 2010), suggesting a direct link between microsaccade activity and visual perception. They can also causally enhance or suppress peripheral visual sensitivity and contrast sensitivity in humans (Hafed 2013; Chen et al. 2015; Wu and Hafed 2025; Zhang et al. 2024).

Thus, the role of microsaccades in fixational eye movement and their relationship with drift still needs to be fully understood. Engbert and Mergenthaler (2006) suggested that microsaccades are triggered by a reduction in drift movement, i.e., low retinal image slip. This idea was further explored by suggesting a relationship between the self-avoiding random walk model and microsaccade triggers (Engbert et al. 2011). Our study provides further evidence supporting this connection. A build-up of activation in the SAW model’s current position is associated with microsaccades, consistent with earlier findings that a decrease in movement precedes these events (Engbert and Mergenthaler 2006). Our data-driven analysis revealed that the activation rises more before a microsaccade and drops more steeply compared to the randomized controls, indicating that high activation levels are more likely to trigger microsaccades. By comparing model variations, we find that this trend is primarily related to the potential, indicating that the portion of microsaccades we capture with our analysis is related to fixation control. However, further investigation is required to determine the exact mechanism behind this relationship and the potential causal direction between activation and microsaccades.

Our modeling approach offers a plausible generative account of the link between drift and microsaccades. It cannot, however exclude alternative explanations and links between microsaccades and drift, e.g. as discussed in (Tian et al. 2016). During high-precision fixation tasks, for example, microsaccades have been found to be corrective of fixation errors most of the time. Further work is needed to disentagle and fully characterize the relationship of drift and microsaccades.

### Other trajectory models

Physiological drift, as the slow component of fixational eye movement, is often modeled as a random walk (Burak et al. 2010; Kuang et al. 2012). Particularly when it is considered mainly as a component in a model of visual processing (e.g., Schmittwilken & Maertens, 2022), this approximation can yield good results. However, the statistical properties of the trajectories do differ significantly from simple randomness. To better capture these aspects, a self-avoiding random walk has been proposed (Engbert et al. 2011; Roberts et al. 2013). However, the number of models that aim to predict fixational movement trajectories is limited. The SAW model used in this paper is one example. Another self-avoiding random walk model was published by (Roberts et al. 2013). Instead of an elliptical activation trace Roberts et al. (2013) implement the self-avoidance by choosing each step direction from a continuous distribution weighted by the density of recent gaze history in each direction. It achieves a similar result: at short time scales, the model is persistent and avoids previously visited areas. The process’s memory is limited and parametrized, allowing the authors insight into the process memory by estimating parameters. Due to the lack of a constraining potential, this model does not represent the subdiffusive component at long time scales.

### Input dependence

Although initially fixational drift was often characterized as noise produced by the oculomotor units (Eizenman et al. 1985), more recent evidence from electrophysiological recordings in monkeys shows that fixational drift originates higher up in the chain of command than the oculomotor neurons (Ben-Shushan et al. 2022) and is influenced by attentional processes (Shelchkova et al. 2019). Microsaccades, too, are influenced by attention and preferentially move in the direction of the attended region when there is covert attention (Hafed and Clark 2002; Engbert and Kliegl 2003). Both microsaccades and drift are also modulated by visual stimuli (Malevich et al. 2020; Khademi et al. 2024). Thus, drift and microsaccades depend on the viewing task and the features of the fixated target (Bowers et al. 2021). This interplay of perception and action is consistent with active vision (Findlay and Gilchrist 2003), even at the scale of fixational eye movement.

The SAW model is stimulus-independent. Other modeling approaches have investigated the interdependence of fixational eye movements and visual perception. It can be shown that the visual processing stream is quite capable of dealing with the hypothesized motion blur caused by the constant displacement of the stimulus over the receptors (Packer and Williams 1992). Fixational drift is beneficial for high-acuity vision, presumably because it allows spatial information to be redistributed into the temporal domain, modulating the input to individual receptors (Clarke et al. 2021). Image-computable models of edge detection can actually be improved by introducing drift (Schmittwilken and Maertens 2022).

Research investigating the role of motion in visual perception typically uses a random walk and is most likely robust to changes in the precise type of motion. However, the experimentally observed statistical properties of drift differ from simple random noise. More research is needed to ascertain whether these properties convey additional benefits to visual processing. A recent paper Anderson et al. (2020) suggested a joint approach to infer movement and stimulus simultaneously. The model assumes a grid of retinal cells onto which stimulus patterns are projected and that the visual processing system cannot access an efference copy of the movement. Instead, they use Bayesian inference to estimate the movement and stimulus from the spike rate generated by the retinal cells alternatingly. The authors conclude that drift is beneficial for high-acuity vision as it helps the system to average over inhomogeneities in the retinal receptors.

Thus, fixational eye movements play an essential role in visual processing. Integrating the fact that it is both stimulus-dependent and individually characteristic suggests that the movement is optimized to account for individual physiological differences. This finding is consistent with the finding that fixational eye movement and visual acuity are related (Clarke et al. 2021). A future direction for fixational drift research may be to implement the idea that drift improves visual acuity in a generative model to infer the ideal motion, prevent fading, or enable edge detection. Furthermore, although visual processing has been found to be quite robust, the development of more accurate models of fixational eye movement may improve the quality of models of visual processing.

There are two considerations that limit the generalizability of the current study. First, the data were recorded using a video-based eye tracker with built-in filtering, which may influence fine-grained features of drift, such as small velocity fluctuations or directional biases. While this does not affect model fitting directly, it may shape some of the observed trajectory details. Second, the study was conducted under sustained fixation, a controlled setting that supports individual-level parameter estimation but does not reflect the dynamics of natural viewing. In more natural contexts, both drift and microsaccades are shaped by task demands, attentional shifts, and stimulus content (Ko et al. 2010; Shelchkova et al. 2019; Poletti et al. 2013; Intoy et al. 2021; Poletti 2023). Extending the model to these conditions may require adapting the potential or incorporating top-down influences.

### Conclusion

In conclusion, our study investigated a dynamical model of fixational eye movements and microsaccades using likelihood-based (Bayesian) parameter inference. Our analyses suggest that self-avoiding random walk models can effectively capture individual fixational drift behavior, as evidenced by the convergence of distinct marginal posteriors for each observer. Furthermore, our data-driven analysis indicates a relationship between microsaccade rate and peaks in the model’s latent activation state, providing further insight into the underlying neurophysiological mechanisms of microsaccade triggering. Overall, our results provide new insights into fixational eye movements and highlight the importance of individual differences in this remarkable behavior.

## Methods

### The likelihood-based modelling framework

Biologically motivated, mechanistic models allow researchers to test whether the proposed mechanisms are capable of producing the observed behavior, to identify which components are essential, and to explore how changes to the system’s structure alter its output (Bechtel and Abrahamsen 2010). Historically, the standard approach for cognitive models involves comparing them to time-independent summary statistics, e.g., here it may be MSD. The likelihood-based approach offers a number of advantages. First, it is possible to estimate the model parameters from the data in a fully Bayesian and statistically rigorous way. The value of the model is independent of any particular ad-hoc metric, the researcher may want to investigate (Schütt et al. 2017). The model likelihood can further be used as a basis for model comparison. Lastly, using the estimated parameters to simulate data, it is possible to conduct posterior predictive checks using metrics such as MSD to investigate whether the data constrains the model in a way that produces the expected behavior (Engbert 2021). Thus, likelihood-based parameter inference allows compelling conclusions about the underlying mechanisms. Another advantage is that Bayesian parameter estimation provides a natural way to quantify uncertainty in the estimates, through the use of posterior distributions and credible intervals. This can be especially useful in cases where the data are noisy or the model is complex.

By independently estimating separate parameters for each experimental subject, it is possible to investigate individual differences. The parameter estimation yields a separate posterior distribution for each subject. As the parameters represent interpretable quantities with biological counterparts, the comparisons of the posteriors can allow interesting insights. Additionally, when a model is capable of representing individual differences, it speaks to the validity of the model and its parametrization.

### Experimental data

The experimental data used for this study were eye movement trajectories recorded using an Eyelink 2 with a sampling rate of 500 Hz. Participants were seated at a distance of 50 cm to the monitor and calibrated using a 9-point calibration grid. Each trial consisted of a fixation task, where participants fixated a cross in the center of a white screen for 3 seconds, followed by a scene viewing task. Here, we use only the enforced fixation data from the first 3 seconds. Out of 50 recruited participants, 48 completed all 40 trials. A further 6 were later excluded due to a large number of blinks. As the experiment included a rigorous online quality control and the calibrate-ability of subjects varies, 2 participants aborted the experiment. Trials during which saccades or blinks were detected were repeated immediately. In order to detect microsaccades we used a velocity-based algorithm (Engbert et al. 2015). The data set is publicly available on the Open Science Framework (www.osf.org/hf2dw)

### Parameter estimation

Here, we used the DREAM_ZS_ algorithm (Laloy and Vrugt 2012). Rooted in the classical Metropolis (Hastings) Markov Chain Monte Carlo (MCMC) algorithm, DREAM_ZS_ iteratively explores the parameter space by sampling its position and asymptotically converges to the true posterior distribution of the parameters. The algorithm includes several (Markov) chains starting at arbitrary (random) positions in the parameter space. For each chain new positions are chosen by combining (hence “evolution”) positions of other randomly chosen chains, including their past states.

**Fig. 8:**
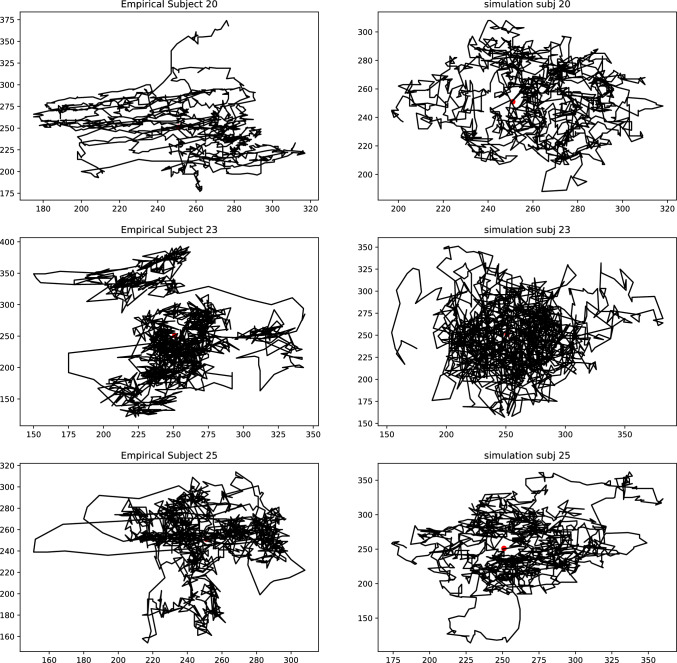
Some examples of fixational eye movement traces. The left column shows the experimental data. The right column shows data simulated using the individually fitted models. These examples qualitatively illustrate the captured statistical properties of the drift movements

**Fig. 9:**
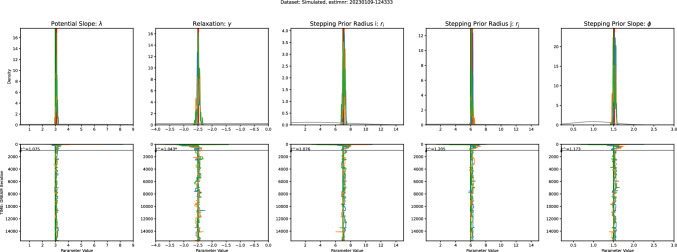
Parameter Recovery Analysis. For each parameter we show that a recovery analysis converges to the correct value. The top row shows the posteriors relative to the priors and reveals strong convergence in all parameters. The bottom row shows the caterpillar plots of the estimation procedure, i.e., the parameter value that the three chains assumed in each iteration. The horizontal line indicated the burn in period

**Table 1:** The parameters that define the prior distributions used during parameter inference. For each parameter we report the mean, standard deviation, as well as the upper and lower bounds of the truncated Gaussians

	Potential Slope: $\lambda$	Relaxation: $\gamma$	Stepping Prior Radius i: $r_i$	Stepping Prior Radius j: $r_j$	Stepping Prior Slope: $\phi$
mean	2	$-$2.5	5	5	1.5
sd	2	0.3	5	5	0.5
lower	0.3	$-$3.8	0.1	0.1	0.5
upper	8	0	15	15	3

In Bayesian parameter estimation, the unknown parameters are treated as random variables and are assigned a prior probability distribution. This prior distribution reflects the researcher’s initial beliefs about the likely values of the parameters based on prior knowledge or experience. We chose relatively broad truncated Gaussian priors, which did not constrain the estimation very strongly. The truncated tails were chosen according to experience with the model to prevent numerical problems in the case of extreme parameter values.

We split this data set into two separate sets: one half (20 subjects) was used for model development and exploratory analyses. The other half (21 subjects) was used for the final analyses and model evaluation. Each set contains data from 27 trials for each subject. We discarded all trials where the movement during fixation exceeded 1.2 degrees of visual angle. Due to the high individual variability in the data this criterion excluded 7 subjects, because too many trials were affected. The 27 trials can be split into training and test sets, with a 2/3 to 1/3 split. Each trial was 1500 samples long, i.e. represented a fixation of 3 seconds. This procedure finally yielded a data set with equal numbers of samples per trial and trials per subject, facilitating statistical analyses.

## Note on comparisons of turning-angle distributions

In order to compare and correlate the distributions turning angles of individual participants as well as between simulated and observed data, it was necessary to reduce the angle distribution to a single summary value. The area under the curve (AUC) metric is a common solution to this problem. However, in our case the distributions are densities, i.e., their AUC was equal to one by definition. Instead, we compare the AUC of the empirical cumulative density function (ECDF). The ECDF is obtained by sorting the observations into unique bins and calculating the cumulative probability for each. By grouping and computing the ECDF AUC of each group, we can compare the similarity in the peak height of the angle distributions.

## Appendix

### Discretization

The model is defined on a $100\times 100$ lattice. In order to evaluate experimental eye movement traces (which are typically given in degrees of visual angle), we discretized the data. Each data point was multiplied by 350 and then applying the floor function. This discretization value for the entire data set was chosen by visual inspection, as it allowed all eye movement traces to stay within the confines of the grid, but also efficiently used the space. Parameter values from the stepping distribution to the potential critically depend on the specifics of this discretization.

### Simulated data

Figure 8 shows some examples of experimental gaze trajectories which illustrate that the model captures individual differences in the data that can be validated by visual inspection.

### Parameter recovery

Parameter recovery analyses are an important step in evaluating the stability and reliability of a mathematical model. This is often done as a first step in model building to ensure that the model is able to accurately capture the underlying dynamics of the system being studied. Parameter recovery analyses involves generating synthetic data with known (“true”) parameter values of the model and then estimating the parameters from this simulated data. This procedure permits the evaluation of the accuracy and precision of the parameter estimates, as well as the sensitivity of the estimates to the choice of estimation method and the quality and amount of data. It is an important step to assess the overall performance of the model and identify potential issues that may arise in the estimation process.

### Priors

For the parameter estimation we chose truncated Gaussian priors. Table 1 details the numerical values that define the truncated Gaussian priors.

### Parameter estimation results

Table 2 gives the detailed point estimates for all estimated parameters for all participants, as well as 98% confidence intervals. Note that the participant IDs start at 20, since we estimated parameters for the final model for participant IDs 20 to 39 only. The data for participant IDs 1 to 19 were used for model building and are omitted here to prevent overfitting.

**Table 2:** Point estimates and 98% confidence intervals of the 5 estimated parameters for each subject

Subject	Potential Slope: $\lambda$		Relaxation: $\gamma$		Stepping Prior Radius i: $r_i$		Stepping Prior Radius j: $r_j$		Stepping Prior Slope: $\phi$	
	mean	+/-	mean	+/-	mean	+/-	mean	+/-	mean	+/-
20	4.969	0.194	$-$3.585	0.209	3.304	0.124	2.664	0.083	1.080	0.021
21	4.497	0.162	$-$3.688	0.110	3.850	0.204	3.378	0.166	1.072	0.032
22	4.917	0.185	$-$3.727	0.072	3.153	0.106	2.333	0.081	1.062	0.028
23	4.533	0.045	$-$3.773	0.027	12.064	0.572	8.181	0.376	1.203	0.055
24	4.796	0.179	$-$3.693	0.104	3.402	0.152	2.644	0.091	1.077	0.030
25	4.563	0.167	$-$3.757	0.043	5.156	0.254	4.172	0.215	1.010	0.032
26	4.646	0.144	$-$3.767	0.033	7.301	0.266	4.562	0.195	1.199	0.026
27	5.100	0.163	$-$3.692	0.105	2.766	0.143	2.171	0.109	0.963	0.031
28	4.940	0.165	$-$3.700	0.098	3.558	0.171	2.152	0.071	1.052	0.035
29	5.211	0.165	$-$3.723	0.076	3.353	0.093	2.700	0.077	1.111	0.022
30	4.778	0.217	$-$3.750	0.050	3.700	0.133	2.193	0.104	0.972	0.028
31	4.621	0.115	$-$3.771	0.028	7.844	0.296	5.613	0.209	1.191	0.031
32	4.504	0.146	$-$3.759	0.041	11.304	0.393	6.701	0.223	1.217	0.039
33	4.565	0.223	$-$3.696	0.103	3.527	0.157	2.499	0.102	1.012	0.028
34	4.922	0.160	$-$3.720	0.080	4.331	0.207	3.204	0.165	1.085	0.037
35	4.693	0.209	$-$3.736	0.061	3.418	0.125	2.502	0.087	1.005	0.021
36	4.708	0.132	$-$3.765	0.035	7.780	0.336	5.351	0.220	1.220	0.037
37	4.223	0.104	$-$3.755	0.044	8.409	0.286	6.516	0.222	1.211	0.030
38	4.868	0.142	$-$3.732	0.067	3.914	0.116	2.751	0.091	1.092	0.025
39	5.061	0.091	$-$3.712	0.084	4.460	0.188	3.176	0.128	1.163	0.035
